# The Surgical and Therapeutic Treatment of Caries and Necrosis of the Alveolar Process in Man

**Published:** 1887-04

**Authors:** A. W. Harlan

**Affiliations:** Chicago, Ill.


### Independent Practitioner.

Vol. VIII. April, 1887. No. 4.

### Original Communications.

Note.—No paper published or to be published in another journal will be accepted for this department. All papers must be in the hands of the Editor before the first day of the month preceding that in which they are expected to appear. Extra copies will be furnished to each contributor of an accepted original article, and reprints, in pamphlet form, may be had at the cost of the paper, press-work and binding, if ordered when the manuscript is forwarded. The Editor and Publishers are not responsible for the opinions expressed by contributors. The journal is issued promptly, on the first day of each month.

### THE SURGICAL AND THERAPEUTIC TREATMENT OF CARIES AND NECROSIS OF THE ALVEOLAR PROCESS IN MAN.

BY A. W. HARLAN, M. D., D. D. S., CHICAGO, ILL.

Read Before the Central Dental Association of Northern New Jersey at Montclair, Jan. 20, 1887.

Caries and necrosis of the alveolar process is observed at all ages of man, more frequently, however, after the age of puberty. If disease attacks the alveolar process disastrously at an early age, the subjects are usually found to be inmates of foundling asylums, workhouses, orphan asylums, or other similar institutions, or in the densely crowded dwelling places of the poor, where infection and contagion float in the air, in the regions where bad drainage, imperfect ventilation, and non-attention to necessary hygienic surroundings is prevalent. Under such conditions, and in the places mentioned, Dental Surgeons rarely come in contact with the sequelæ of exanthematous and other acute diseases, and hence are usually unfamiliar with the appearance of mouth lesions of the soft or bony structures in early childhood in eleemosynary institutions or the abiding places of poverty and filth. It is questionable, therefore, whether the statement is true that diseases of the alveolar process are more prevalent after the fourteenth year on account of the inferences to be drawn from the above. At any rate the dentist comes in contact with more cases of alveolar disease after the patient has passed the age of puberty than before it is attained.

In a certain sense the proper treatment of diseased alveolar processes implies a thorough understanding of the causes of disease, yet in many instances this is untrue, for who has not observed cases, after recovery, where little if any systematic treatment had been employed, and no deformity existed in consequence thereof? This only goes to show the wonderful reparative process of nature. What are the usual causes of disease in the oral cavity which affect so injuriously the bony structure surrounding teeth? One principal cause is disease resulting from the death of the pulp; others from syphilis, the exanthemata, phosphorus, arsenic, excessive wedging, and other traumatisms, bungling regulating apparatus, salivary concretions, ostitis, periostitis, osteomyelitis, ill-fitting partial dentures, sepsis, and numerous ills which every one can catalogue to make his summary complete. The death of the pulp, its subsequent decomposition and abortive attempts to remove it, and the later unsuccessful efforts to fill the root, may be reckoned as the principal causes of caries of bone when ignorance or neglect of proper treatment results in acute inflammations with their sequences. An alveolar abscess is never found at the apex of a root, or leading from it, unless there be a coincident excavation of bone around it, or a perforation of the process in its near proximity. Occasional rare cases of perforation of the antral cavity, burrowing of pus into other regions, as on the neck, the chin, the nasal cavity—between the palate bones, or even through the lower maxillary—have been reported with concomitant caries of bone, and they occur in time in the practice of almost every one. Still the greater number of cases of caries observed are found in the neighborhood of the affected teeth. In chronic abscesses from pulpless teeth a gradual but slow solution, or breaking down of the margins of the process making the outline of the original destruction is observed. In time this may be so marked that pressure of the finger will bring the gum in contact with the root. The edges of the alveolar process in such cases are not necrosed; they are simply carious. Ultimately such teeth, without treatment, become useless, and either drop out or are extracted on account of the destruction of the pericementum and their consequent feeble attachments to the alveoli. Many teeth with excellent fillings in them are left by operators to discharge pus into the mouth daily, either through ignorance of the requisite treatment, carelessness, or timidity about operating surgically. There are many more cases of caries of bone around the roots of teeth than there are of necrosis. You may ask, Why is this so? or you may dispute it. In searching for the reasons to substantiate this statement, we may say that the ignorance of the average dentist is to be deplored, especially when speaking of the methods of removal of the pulp, and the filling of roots of teeth. No authoritative manual of operating in either of these procedures has ever been published. It is impossible to enumerate the various instruments used, and the methods of practice pursued in removing this delicate organ. You are well aware of the fact that no universal root-filling material is used. When it is remembered that roots of teeth are filled with every known plastic, cotton, gold, lead, wood, silk, and combinations of antiseptics too numerous to prove of value, is it not wonderful that we do not find even a larger number of chronic abscesses than are seen? In my short professional life I have visited many dental offices, and from what I have observed I am no longer surprised that portions of the pulp are left to putresce in the roots of teeth, or that roots are often imperfectly filled. When you have not the necessary instruments it is impossible to perfectly remove the pulp. This is also true when applied to the filling of roots. In this connection I desire to state emphatically that the practice of soaking wood, cotton and silk, or incorporating antiseptics with paraffine, shellac, gutta percha or other plastics, in the hope that if the filling does not reach the apex of a root, the antiseptic will forever remain as a guard against putrefaction, is delusive, unscientific, and demonstrates the lack of a respectable knowledge of the lasting property of an antiseptic. Such juggling as this needs, as it deserves, the severest condemnation. With such sentiments and convictions it is very easy to detail a line of treatment for caries of the alveolar process in man. Caries of bone, from whatever cause arising, is to be treated surgically, by dividing the gum in any approved manner, and boring into the apicial space with clean instruments, and under antiseptic precautions. The cavity being cleansed and packed with antiseptic dressings, which are to be replaced with balsamic or non-irritating coverings as new tissue is developed to fill the space once whole. The prophylaxis is proper removal of the pulp, and a well-defined system of root filling with a material non-metallic, innocuous, and well adapted for the purpose—gutta percha. In dismissing this portion of the subject, the writer is aware that many teeth develop abscesses around their roots when unfilled, and indeed caries of the process is observable from inflammations of the gingivæ and other causes around teeth containing living pulps, as in phagedenic pericementitis, or pyorrhœa alveolaris; but it is not the intention to dwell on this latter phase of disease, only in so far as to say that the diseased bone should be removed with the same precautions, but the subsequent treatment differs, as it may involve sponge-grafting, the use of astringents, local stimulants, germicides or disinfectants. Tents of cotton, felt or gauze packed between the gums and the teeth are rarely requisite pending the reformation of tissue from such trivial operations, although the importance of correct treatment of such conditions is even greater than in the aforementioned. Necrosis of the alveolar process, although less frequently met with than caries, is the *bete noir* of the every-day practitioner. Phosphor-necrosis is very rare at the present time, and the details of treatment are to be found in the works of Heath, Garretson, and works on general surgery, and consequently I will not burden you with a repetition. Necrosis of the alveolar process at times has followed very trivial causes. By necrosis is meant death of the bone *en masse.* I have seen a well-built woman, weighing one hundred and sixty pounds, reduced to a pitiable state of nervousness, losing more than thirty pounds in weight in ten days, from the forcing of septic matter through the root of a bicuspid tooth. The outer plate of the alveolar process from the first molar to the median line became necrosed from the retention of pus in contact with the bone and in due time it was removed without loss of a single tooth. It may be stated as a general proposition that danger to health, and even to life, is always invited by irresolution or timidity in operating promptly in the beginning of peridental inflammation, which has its origin in or near the apicial space. It is undoubtedly true that there are many cases of necrosis of the alveolar process not due to causes arising from the death of the pulp. However, many more are seen whose origin can be traced directly to this cause. Nearly every case of this kind occurring in the mouths of children after the sixth or seventh year is due to defective teeth. Mercury and syphilis were formerly held to be the cause of nearly every disease which the surgeon or physician did not understand, but now things are changed. It matters not whether necrosis be due to traumatism, sepsis, microbes, an imperfectly-filled root, an impacted tooth, or faulty adjustment of a splint. We must discover the cause, not so much for the aid it will afford for treatment, but to prevent future pain, discomfort or deformity for others. Preventive medicine is what we are aiming at. To become dental officers of health requires sound judgment, much study, and accurate observation. Can we expect to escape the penalties due to ill-advised operations on pulpless or other teeth in the mouths of the growing young, enfeebled, the aged, or those whose time is of so much value that they cannot afford to submit to proper treatment? The operator who only sees present gain at the finish is too dishonest to practice a beneficent calling. Scientific acquirements, great learning, skill and fearlessness are of little value unless joined to honesty of purpose. Ignorance of methods, or of the causes of disease is no excuse for neglect of proper treatment; but honesty in practice, added to a desire for knowledge, will conquer the most desperate case, because such devotees will counsel with their confrères and bring about a successful issue. You may ask what this has to do with the treatment of necrosed bone, but your own intelligence will answer it because my plea is for a thorough study of the causes of disease, a correct diagnosis, and prognosis, the certain method of operating, a scientific treatment based on these, and above all prophylaxis. All these preliminaries have been gone over to show the necessity for preventing the death of the pulp, but if it dies or is destroyed intentionally, the greatest care and skill must be exercised to prevent abscess and possible necrosis. If, unfortunately, some vital step has been overlooked or neglected, and we are brought to face the inevitable sequel of such neglect, the necessity for promptness is only too apparent. Good drainage is the first consideration, after which sustention of the vital forces; these having been looked after, cleanliness and consecutive attention to details follows. It is often unwise to remove portions of necrosed bone, even though irritating to the soft tissues, until the teeth are likely to be retained in place by new formations. It is better practice, as a rule, to allow the sequestrum to be taken away in fragments rather than invite a loss of teeth, or even more living useful bone.

Dr. Atkinson, in a paper read before the section on diseases of the teeth, International Medical Congress, London, 1881, says: "The prevailing methods in surgery of treating caries and necrosis by expectant treatment and drainage should no longer be resorted to. The portions already dead, or greatly debilitated, should be thoroughly removed well up to the healthy territory—securing a pocket to receive the pabulum, out of which to attain reproductions by what has been called 'first intention.'" This teaching is founded on correct surgical principles, although in the treatment of some cases I might be inclined to doubt the propriety of at once removing *all* dead bone around teeth in beginning the treatment of a case, as has previously been stated. The attempt should be made in all cases to save useful teeth by wiring them or by the adjustment of a metallic perforated skeleton splint, which will securely hold them in place until the new formations are sufficiently dense to maintain them in position unaided. This is a matter of great importance.

SPECIAL THERAPEUTICS.

In the treatment of caries or necrosis of the bone little need be added to the foregoing. Methods of practice are varied to suit the case, and the remedies used or proposed from time to time are sufficiently numerous to meet the wants of the most exacting. The principles of local treatment may be summarized as follows: Premising that the root has been filled if indicated, the surgical wound may be cleansed with peroxide of hydrogen, a ten per cent. aqueous solution of resorcin, a three per cent. solution of aseptol, or equal parts of a one in one thousand solution of corrosive sublimate and $H_2O_2$. Any of the above named are efficient, and of nearly equal value for this purpose. The wound should then be packed with cotton sprinkled with deodorized iodoform (for this, use crystals of thymol, finely powdered coffee or oil of cinnamon three drops to ℥ ss.), or the cotton may first be moistened with pure tereben. As a substitute, use iodol dissolved in hot oil (ethereal or a fixed oil), or dip the cotton in tereben, and powder the surface with iodol. These dressings need not be changed daily. If extraneous matters have been kept from the wound it must not be irrigated with escharotic or irritating lotions. If at any time it is deemed unwise to remove the whole sequestrum in necrosis, the parts must be kept clean by the daily use of any of the above-named cleansing and stimulating agents, the dressings only being indicated after operations on carious or necrosed bone, until complete restoration of the parts has resulted. It will be noted that in some respects the therapeutic treatment laid down is not in accord with the teaching of the books, but this is the age of progress, and progress is due largely to gatherings of this kind, where all that is illogical, absurd, or misleading in theory or practice is quickly discerned, and the stamp of approval is only affixed when truth is disclosed, unshadowed by superstition or the false logic of preconceived ideas.

